# Supplementary material for: Predicting the taxonomic and environmental sources of integron gene cassettes using structural and sequence homology of attC sites
Source: Commun Biol. 2021 Aug 9;4:946. doi: 10.1038/s42003-021-02489-0 (PMC8352920; doi:10.1038/s42003-021-02489-0)
Supplement: Supplementary file 2 — Supplementary Information [file 42003_2021_2489_MOESM2_ESM.pdf]

Supplementary Tables and Figures

Supplementary Table 1. Efficacy of covariance models for *attC* taxonomy predictions

**Taxon of covariance model**	**# *attC*s**	**Bit score cut-off**	**Sensitivity (%)**	**Specificity (%)**
Alteromonadales (Gammaproteobacteria)	129	62	31.0	99.9
Methylococcales (Gammaproteobacteria)	448	63	71.7	99.6
Oceanospirillales (Gammaproteobacteria)	82	79	61.0	100.0
Pseudomonadales (Gammaproteobacteria)	83	60	50.6	99.8
Vibrionales (Gammaproteobacteria)	248	45	99.2	99.7
Xanthomonadales (Gammaproteobacteria)	505	73	98.4	98.0
Acidobacteria	120	64	30.0	99.6
Cyanobacteria	51	61	60.8	99.8
Deltaproteobacteria	72	64	68.1	100.0
Planctomycetes	188	60	71.8	99.3
Spirochaetes	52	43	84.6	99.9

Supplementary Table 2. Bacterial orders that contain *attC*s belonging to the Xanthomonadales-like clade

**Order**	**# Genomes**	**# *attC*s**
Xanthomonadales	54	935
Burkholderiales	1	3
Methylococcales	1	3

Supplementary Table 3. Bacterial genera that contain *attC*s belonging to the Xanthomonadales-like clade

**Genus**	**Order**	**# Genomes**	**#*attC*s**	***attC*s per genome**
Xanthomonas	Xanthomonadales	32	282	8.8
Lysobacter	Xanthomonadales	8	270	33.8
Luteimonas	Xanthomonadales	5	144	28.8
Pseudoxanthomonas	Xanthomonadales	6	132	22.0
Thermomonas	Xanthomonadales	2	102	51.0
Stenotrophomonas	Xanthomonadales	1	5	5.0
Comamonas	Burkholderiales	1	3	3.0
Methylocaldum	Methylococcales	1	3	3.0

Supplementary Figure 1. Structure-based clustering of *attC*s. This tree represents the same tree displayed in Fig. 2 of the article, except with all branches labelled. Included are the ten top-scoring (based on covariance model (CM) bit scores) *attC*s for each taxon-specific CM as well as the *attC* sites from 108 different resistance gene cassettes annotated by Partridge et al. (10). Each resistance cassette *attC* is labelled by the gene name and example accession provided by Partridge et al. (10).

Supplementary Figure 2. A maximum-likelihood tree inferring the phylogeny of integron integrases (IntI). Marine species are shown in blue text, while those from soil/freshwater environments are shown in brown text. IntI phylogeny clusters according to environment forming two major clades, a marine clade (blue shaded region) and a soil/freshwater clade (brown shaded region). Inverse integrases form a sub-clade outlined by the black dotted lines.

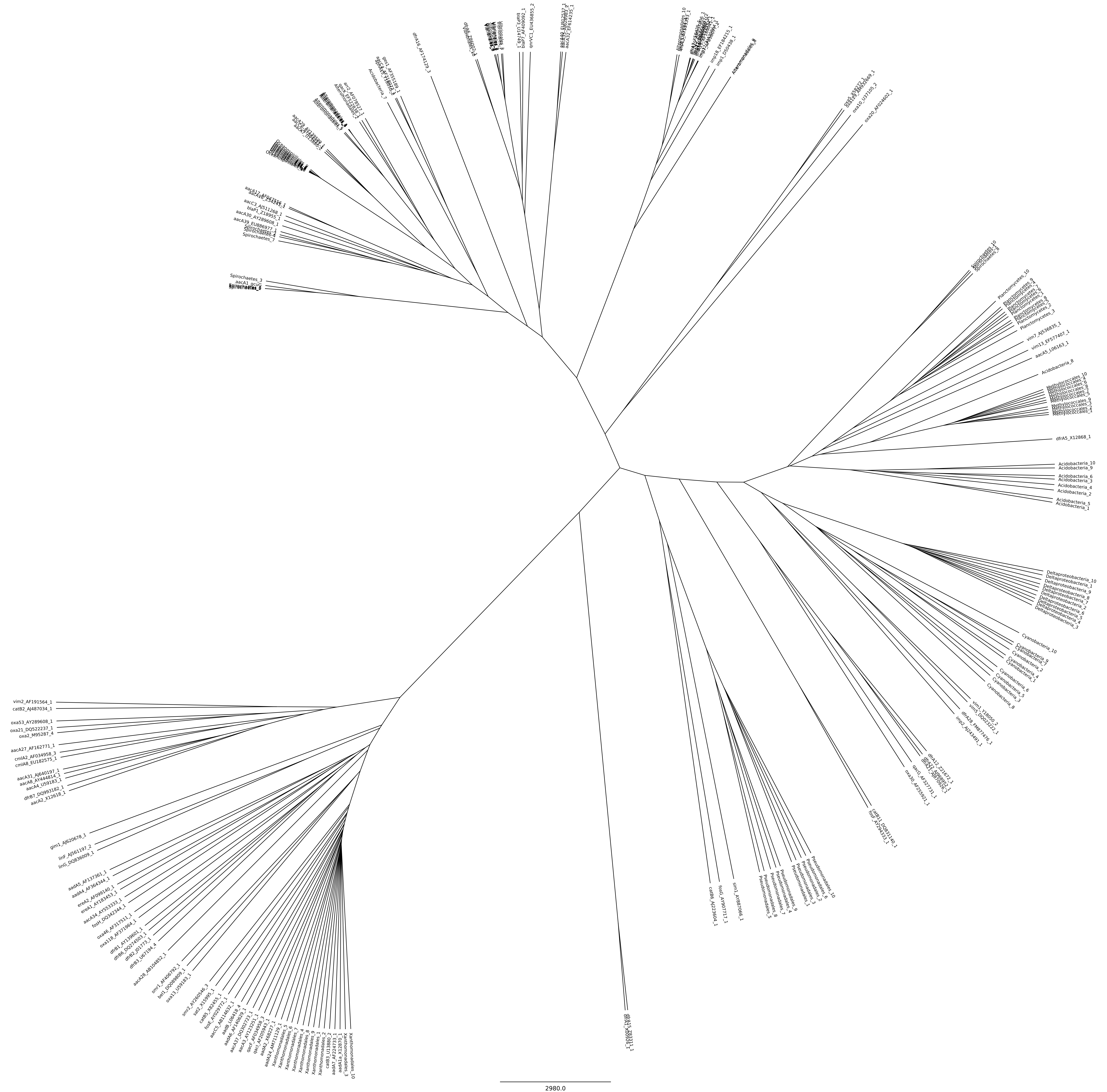

vim2_AF191564_1
catB2_AJ487034_1
oxa53_AY289608_1
oxa21_DQ522237_1
oxa2_M95287_4
aacA27_AF162771_1
cmlA2_AF034958_3
cmlA8_EU182575_1
aacA31_AJ640197_1
aacA8_AY444814_1
aacA4_U59183_1
dfrB7_DQ993182_1
aacA2_X12618_1
gim1_AJ620678_1
linF_AJ561197_2
linG_DQ836009_1
aadA5_AF137361_1
aadA4_AF364344_1
ereA2_AF099140_1
ereA1_AY183453_1
aacA34_AY553333_1
fosH_DQ342344_1
oxa46_AF317511_1
oxa118_AF371964_1
dfrB1_AY139601_1
dfrB6_DQ274503_1
dfrB2_J01773_1
dfrB3_U67194_4
aacA28_AB104852_1
smr1_AF406792_1
bel1_DQ089809_1
oxa13_U59183_1
smr2_AY260546_3
sat2_X15995_1
catB5_X82455_1
fosE_AY029772_1
aacC5_AB114632_1
aadB_L06418_4
aadA6_AF140629_1
aacA37_DQ302723_1
aacA3_AY123251_1
qacF_AF034958_3
qacI_AF205943_1
aadA2_X68227_1
aadA24_AM711129_1
Xanthomonadales_5
Xanthomonadales_6
Xanthomonadales_4
Xanthomonadales_8
Xanthomonadales_1
Xanthomonadales_9
Xanthomonadales_2
catB3_U13880_2
aadA7_AF224733_1
aadA1a_X12870_1
Xanthomonadales_3
Xanthomonadales_10
dfrA1_X00926_1
catB6_AJ223604_1
fosG_AY907717_1
sim1_AY887066_1
Pseudomonadales_5
Pseudomonadales_8
Pseudomonadales_7
Pseudomonadales_9
Pseudomonadales_4
Pseudomonadales_1
Pseudomonadales_3
Pseudomonadales_2
Pseudomonadales_6
Pseudomonadales_10
catB11_DQ831140_1
fosF_AY294333_1
oxa30_AF255921_1
qacG_AF327731_1
dfrA12_Z21672_1
vim1_Y18050_2
vim5_DQ023222_1
dfrA26_FM877476_1
imp2_AJ243491_1
Cyanobacteria_10
Cyanobacteria_9
Cyanobacteria_2
Cyanobacteria_4
Cyanobacteria_1
Cyanobacteria_6
Cyanobacteria_5
Cyanobacteria_3
Cyanobacteria_8
Deltaproteobacteria_10
Deltaproteobacteria_1
Deltaproteobacteria_9
Deltaproteobacteria_8
Deltaproteobacteria_7
Deltaproteobacteria_2
Deltaproteobacteria_6
Deltaproteobacteria_5
Deltaproteobacteria_4
Deltaproteobacteria_3
Acidobacteria_10
Acidobacteria_9
Acidobacteria_6
Acidobacteria_3
Acidobacteria_4
Acidobacteria_2
Acidobacteria_5
Acidobacteria_1
dfrA5_X12868_1
Methylococcales_10
Methylococcales_9
Methylococcales_1
Acidobacteria_8
aacA5_L06163_1
vim13_EF577407_1
vim7_AJ536835_1
Planctomycetes_10
Planctomycetes_3
oxa20_AF024602_1
oxa10_U37105_2
imp18_EF184215_1
imp1_D50438_1
Alteromonadales_8
qnrVC1_EU436855_2
blaP3_U14749_1
blaP7_AF409092_1
dfrA16_AF174129_3
Acidobacteria_7
aacA17_AF047556_1
aacC3_AJ511268_1
blaP1_Z18955_1
aacA30_AY289608_1
aacA39_EU886977_1
Spirochaetes_1
Spirochaetes_7
Spirochaetes_3
aacA1_gcuG
Spirochaetes_6
2980.0

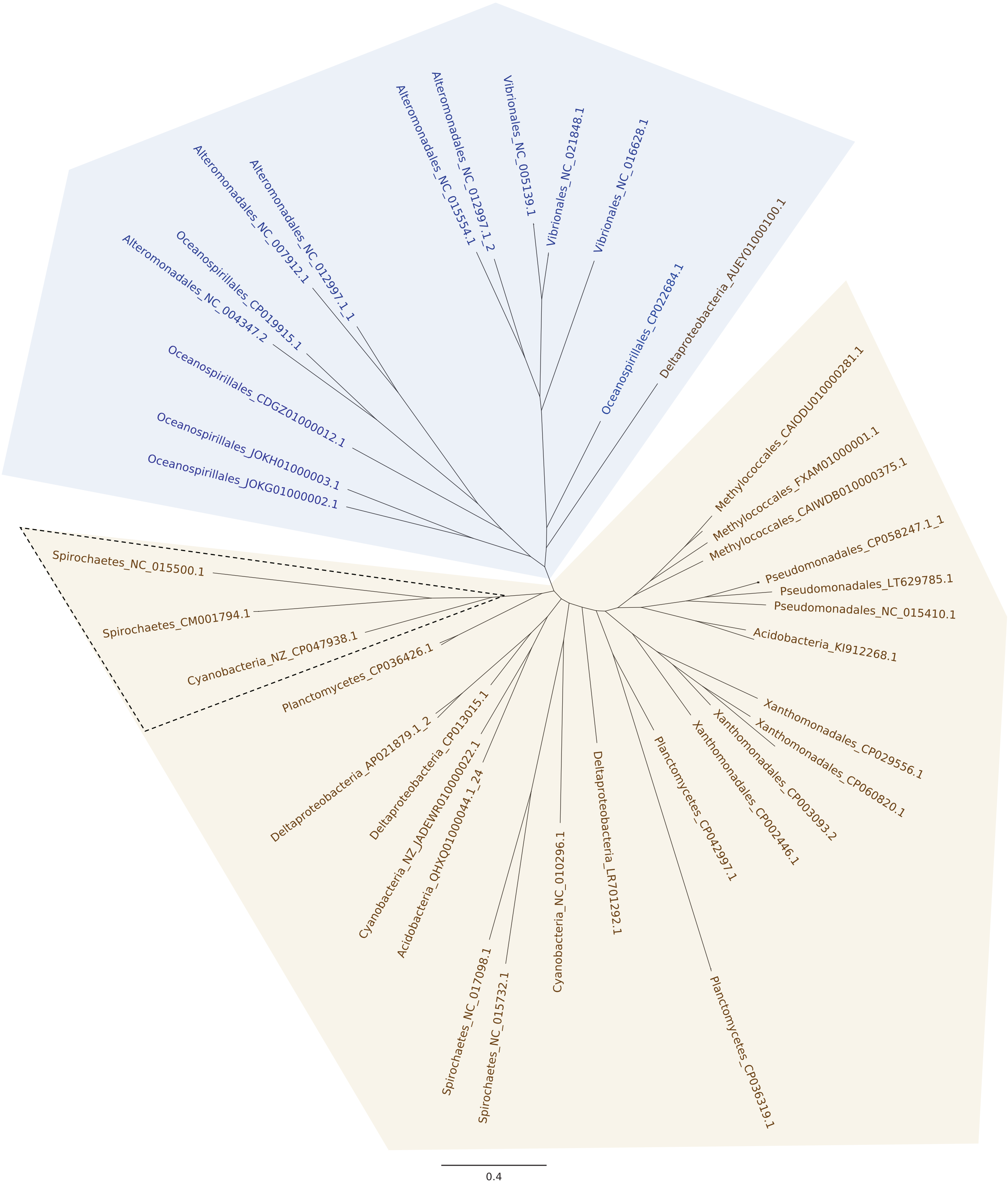

Vibrionales_NC_005139.1
Vibrionales_NC_021848.1
Vibrionales_NC_016628.1
Alteromonadales_NC_012997.1_2
Alteromonadales_NC_015554.1
Alteromonadales_NC_012997.1_1
Alteromonadales_NC_007912.1
Alteromonadales_NC_004347.2
Oceanospirillales_CP019915.1
Oceanospirillales_CDGZ01000012.1
Oceanospirillales_JOKH01000003.1
Oceanospirillales_JOKG01000002.1
Oceanospirillales_CP022684.1
Deltaproteobacteria_AUEY01000100.1
Methylococcales_CAIODU010000281.1
Methylococcales_FXAM01000001.1
Methylococcales_CAIWDB010000375.1
Pseudomonadales_CP058247.1_1
Pseudomonadales_LT629785.1
Pseudomonadales_NC_015410.1
Acidobacteria_KI912268.1
Xanthomonadales_CP029556.1
Xanthomonadales_CP060820.1
Xanthomonadales_CP003093.2
Xanthomonadales_CP002446.1
Planctomycetes_CP042997.1
Planctomycetes_CP036319.1
Deltaproteobacteria_LR701292.1
Cyanobacteria_NC_010296.1
Spirochaetes_NC_015732.1
Spirochaetes_NC_017098.1
Acidobacteria_QHXQ01000044.1_24
Cyanobacteria_NZ_JADEWR010000022.1
Deltaproteobacteria_CP013015.1
Deltaproteobacteria_AP021879.1_2
Planctomycetes_CP036426.1
Cyanobacteria_NZ_CP047938.1
Spirochaetes_CM001794.1
Spirochaetes_NC_015500.1
0.4
