# Supplementary material for: Predicting the taxonomic and environmental sources of integron gene cassettes using structural and sequence homology of attC sites
Source: Commun Biol. 2021 Aug 9;4:946. doi: 10.1038/s42003-021-02489-0 (PMC8352920; doi:10.1038/s42003-021-02489-0)
Supplement: Supplementary file 3 — Description of Supplementary File [file 42003_2021_2489_MOESM3_ESM.pdf]

Description of Additional Supplementary Files

File name: Supplementary Data 1
Description: attC covariance models.
