# Supplementary material for: Predicting the taxonomic and environmental sources of integron gene cassettes using structural and sequence homology of attC sites
Source: Commun Biol. 2021 Aug 9;4:946. doi: 10.1038/s42003-021-02489-0 (PMC8352920; doi:10.1038/s42003-021-02489-0)
Supplement: Supplementary file 5 — Reporting Summary [file 42003_2021_2489_MOESM5_ESM.pdf]

nature research

Corresponding author(s): *Double-blind peer review submissions: write DBPR and your manuscript number here instead of author names.*

Last updated by author(s): *YYYY-MM-DD*

Reporting Summary

Nature Research wishes to improve the reproducibility of the work that we publish. This form provides structure for consistency and transparency in reporting. For further information on Nature Research policies, see our Editorial Policies and the Editorial Policy Checklist.

Statistics

For all statistical analyses, confirm that the following items are present in the figure legend, table legend, main text, or Methods section.

n/a	Confirmed	
☒	☐	The exact sample size (*n*) for each experimental group/condition, given as a discrete number and unit of measurement
☒	☐	A statement on whether measurements were taken from distinct samples or whether the same sample was measured repeatedly
☒	☐	The statistical test(s) used AND whether they are one- or two-sided<br>*Only common tests should be described solely by name; describe more complex techniques in the Methods section.*
☒	☐	A description of all covariates tested
☒	☐	A description of any assumptions or corrections, such as tests of normality and adjustment for multiple comparisons
☒	☐	A full description of the statistical parameters including central tendency (e.g. means) or other basic estimates (e.g. regression coefficient) AND variation (e.g. standard deviation) or associated estimates of uncertainty (e.g. confidence intervals)
☒	☐	For null hypothesis testing, the test statistic (e.g. *F*, *t*, *r*) with confidence intervals, effect sizes, degrees of freedom and *P* value noted<br>*Give P values as exact values whenever suitable.*
☒	☐	For Bayesian analysis, information on the choice of priors and Markov chain Monte Carlo settings
☒	☐	For hierarchical and complex designs, identification of the appropriate level for tests and full reporting of outcomes
☒	☐	Estimates of effect sizes (e.g. Cohen's *d*, Pearson's *r*), indicating how they were calculated

Our web collection on statistics for biologists contains articles on many of the points above.

Software and code

Policy information about availability of computer code

Data collection	Specific bacterial genomes were downloaded from NCBI via accessions provided by Cury et al. Nucleic Acids Res. 2016. 44:45359-4550 using the following Perl command [perl -e 'use LWP::Simple;getstore("http://eutils.ncbi.nlm.nih.gov/entrez/eutils/efetch.fcgi?db=nucleotide&rettype=fasta&retmode=text&id=".join(",",qw(<space-separated list of nucleotide accession numbers>)),"output_filename.fasta");'<br>The remaining DNA sequences were downloaded directly from the NCBI Assembly database (https://www.ncbi.nlm.nih.gov/assembly).
Data analysis	LocARNA v1.9.2.1; HattCI v1.0b; Infernal v1.1.2; CD-HIT v4.6; BLAST v2.7.1; RNAclust v1.3; Prodigal v2.6.3; HMMER v3.2; IntegronFinder v1.5.1; MAFFT v7.271; IQ-TREE v1.6.12; ViennaRNA v2.0.<br>Specific parameters used for each software are provided in detail in the Methods section.

For manuscripts utilizing custom algorithms or software that are central to the research but not yet described in published literature, software must be made available to editors and reviewers. We strongly encourage code deposition in a community repository (e.g. GitHub). See the Nature Research guidelines for submitting code & software for further information.

Data

Policy information about availability of data

All manuscripts must include a data availability statement. This statement should provide the following information, where applicable:

- Accession codes, unique identifiers, or web links for publicly available datasets
- A list of figures that have associated raw data
- A description of any restrictions on data availability

The study has not generated any new DNA sequence data. The data used were either downloaded from the NCBI Assembly database (https://www.ncbi.nlm.nih.gov/assembly) or from the NCBI Nucleotide database using the above Perl command.

The covariance models generated in this study are provided in the supplementary materials.

Field-specific reporting

Please select the one below that is the best fit for your research. If you are not sure, read the appropriate sections before making your selection.

☐ Life sciences ☐ Behavioural & social sciences ☒ Ecological, evolutionary & environmental sciences

For a reference copy of the document with all sections, see nature.com/documents/nr-reporting-summary-flat.pdf

Ecological, evolutionary & environmental sciences study design

All studies must disclose on these points even when the disclosure is negative.

Study description	In this study, we modeled the conserved sequence and structure of integron gene cassette recombination sites (attCs) among distinct bacterial taxa. We used these models to predict the taxonomic and environmental sources of resistance gene cassettes that are now spread by mobile integrons.
Research sample	Downloaded genome sequences from the NCBI GenBank database.
Sampling strategy	N/A
Data collection	Chromosomal sequences were downloaded from the NCBI GenBank database
Timing and spatial scale	Chromosomal sequences for attC screening were downloaded from NCBI in December 2020.
Data exclusions	Sequences representing plasmids were removed from analysis. This was done to limit the analysis to chromosomal integrons.
Reproducibility	This is not an experimental study, thus reproducibility is not an applicable aspect. Nevertheless, all steps of the analyses are described in detail (including all command-line parameters used for all software) and all data used are publicly available
Randomization	N/A
Blinding	N/A

Did the study involve field work? ☐ Yes ☒ No

Reporting for specific materials, systems and methods

We require information from authors about some types of materials, experimental systems and methods used in many studies. Here, indicate whether each material, system or method listed is relevant to your study. If you are not sure if a list item applies to your research, read the appropriate section before selecting a response.

Materials & experimental systems

n/a	Involved in the study	
☒	☐	Antibodies
☒	☐	Eukaryotic cell lines
☒	☐	Palaeontology and archaeology
☒	☐	Animals and other organisms
☒	☐	Human research participants
☒	☐	Clinical data
☒	☐	Dual use research of concern

Methods

n/a	Involved in the study	
☒	☐	ChIP-seq
☒	☐	Flow cytometry
☒	☐	MRI-based neuroimaging
